# Universality of Dynamical Symmetries in Chaotic Maps

**DOI:** 10.3390/e26110969

**Published:** 2024-11-12

**Authors:** Marcos Acero, Sean Lyons, Andrés Aragoneses, Arjendu K. Pattanayak

**Affiliations:** 1 Department of Physics and Astronomy, Carleton College, Northfield, MN 55057, USA; acerom@carleton.edu (M.A.); lyons@carleton.edu (S.L.); arjendu@carleton.edu (A.K.P.); 2 Department of Physics, Whitman College, Walla Walla, WA 99362, USA

**Keywords:** permutation entropy, ordinal patterns, dynamical symmetries, chaos, iterative maps

## Abstract

Identifying signs of regularity and uncovering dynamical symmetries in complex and chaotic systems is crucial both for practical applications and for enhancing our understanding of complex dynamics. Recent approaches have quantified temporal correlations in time series, revealing hidden, approximate dynamical symmetries that provide insight into the systems under study. In this paper, we explore universality patterns in the dynamics of chaotic maps using combinations of complexity quantifiers. We also apply a recently introduced technique that projects dynamical symmetries into a “symmetry space”, providing an intuitive and visual depiction of these symmetries. Our approach unifies and extends previous results and, more importantly, offers a meaningful interpretation of universality by linking it with dynamical symmetries and their transitions.

## 1. Introduction

Chaotic systems are deterministic in nature although they can include a significant amount of embedded noise. They frequently present dynamics that cannot be distinguished from randomness with traditional techniques [1,2,3,4]. Nevertheless, underneath the apparent randomness, they can contain dynamical constraints related to internal temporal symmetries. Recent efforts have unveiled hidden dynamical symmetries using the time series of chaotic systems, including by quantifying temporal correlations among consecutive values of a measurable variable. Permutation entropy [5], for example, uses the relative preponderance of ordinal patterns (also known as words) to quantify the entropy of a time series. Dispersion entropy [6,7] captures both amplitude and order information, making it sensitive to variations in magnitude. Improved permutation entropy [8] incorporates additional statistical properties of the time series to improve robustness against noise. The Horizontal Visibility Graph [9] transforms a time series into a graph based on visibility criteria, revealing temporal structures through graph properties.

Other techniques explore the temporal correlations and dynamical constraints among consecutive events in a time series by combining words based on their natural symmetries [10,11,12,13,14] to discover other details about the dynamics, including uncovering specific dynamical constraints or generalized symmetries.

In this context, an entropy sub-channel representation and analysis [15] has been shown to allow a compact visual representation of system behavior, including changes with parameters, in a space where the location explicitly encodes the dynamical complexity and symmetry of the dynamics. Specifically, when applied to continuous time series generated from the paradigmatic Duffing oscillator, this traces transitions from and out of chaos via symmetry-related families of periodic orbits. As such, this representation distinguishes different kinds of periodicity and chaos and allows us to better understand transitions between these regimes for a given system. In this paper, we use this technique in a variety of 1D maps to map families of chaos (previously revealed with other techniques), and find that this entropy sub-channel representation allows us a compact and clear mapping of transitions between dynamical regimes. In particular, we find a universality in dynamical behavior across a wide variety of maps that is easily displayed in this space of dynamical symmetries. Below, we lay this out in some detail, starting with defining our techniques in the next section before presenting the results and closing with a short discussion.

## 2. Word Populations, Complexity Quantifiers, and Universality

Permutation entropy (PE) is a complexity quantifier based on temporal correlations among consecutive events in a time series. It is computed based on the probabilities of some ordinal patterns (or words) composed of properties of events in a time series. Using PE and the probabilities of the words necessary to compute it, Parlitz et al. [10] classified time series from heartbeats and were able to distinguish patients suffering from congestive heart failure from a healthy group studying beat-to-beat time series, for example.

Based on the words used to compute permutation entropy, different research has focused on identifying the symmetries within these words. Bandt [11] combined words of dimension three and defined some order combination functions, up-down scaling, up-down balance, persistence, and rotational asymmetry, to identify symmetries in the dynamics and applied it to heart and brain activity data to classify them. Gunther et al. [12] introduced alternative combinations of words and the deviations among them to identify dynamical regimes and anticipate changes in dynamics in the Duffing oscillator.

Representing pairs of these quantifiers in a two-dimensional plane has shown to be a powerful tool in classifying complex dynamics and unveiling signals of universality. In particular, the Fisher–Shannon plane [16,17,18], which portrays the Fisher Information Measure versus entropy, was used by Wang and Shang [19] to analyze financial time series and distinguish three different groups based on their behavior. Identifying constraints in the probabilities of the words for chaotic systems, Spichak and Aragoneses [14] were able to put bounds on the Fisher–Shannon complexity plane of these dynamical systems. They were also able to identify different families of chaos. Nguyen et al. [13] introduced TARDYS quantifiers to identify temporal symmetries based on a photonic neuron. They applied the new quantifiers to chaotic maps and identified different chaotic behaviors with the same Lyapunov exponents. They also were able to forecast regular-to-chaos transitions. This work combined the TARDYS quantifiers with permutation entropy to identify different families of chaos in the logistic map. They found equivalent results as those found previously using the Fisher–Shannon plane computed with words [14]. This work also found underlying universality patterns in non-invertible chaotic iterative maps.

These approaches aim at identifying the symmetries underneath the complex dynamics by focusing on the temporal correlations of the words. Nevertheless, the possible symmetries are limited by the dimension *D* of the words. For a more detailed study, one could attempt at finding the symmetries for higher dimensions of the words. This would lead to more richness of the temporal structures, but also present itself as less intuitive to interpret. Yet, because the possible dynamical symmetries of dimension three are limited, each one of these symmetry quantifiers can be thought as a cross section of a higher dimensional symmetry space.

### 2.1. Words Populations

Given a time series $\left\{x_{i}\right\}$, we compute the words of dimension *D* by comparing *D* consecutive values, $\left[x_{i},x_{i+1},x_{i+2},\ldots ,x_{i+D-1}\right]$, where these $x_{i}$ could be raw observational data or the spacing or magnitude of an event (such as a peak or zero-crossing). For dimension D=3, the smallest value is assigned a label 0, the intermediate value a label 1, and the largest value a label 2. For example, if $x_{i}<x_{i+1}<x_{i+2}$, we assign the word 012 and if $x_{i}<x_{i+2}<x_{i+1}$, we assign the word 021, and so on. There are D! words of dimension *D*. By this procedure, we transform the time series of values into a time series of words. We then calculate the population, or probability, of each word in the time series. For a purely stochastic process, we would expect all probabilities to be equal ($P_{1}=P_{2}=\ldots =P_{6}=\frac{1}{6}$). For a different distribution of populations, we can conclude that the dynamics is deterministic, and different hierarchies of word probabilities correlate with different kinds of deterministic dynamics, including with their intrinsic dynamical symmetries.

Word populations capture temporal correlations of the dynamics of the system. However, the ordinalization loses part of the information as it only includes the relative magnitudes of consecutive values (the sequence 100–200–150 is associated the same word $w_{021}$ as the sequence 140–200–190, for example). Nevertheless, this procedure acts as a filter that is robust to experimental noise. Because of this feature of the words, we can only talk about approximate dynamical symmetries in what we can learn from these populations.

**Figure 1:**
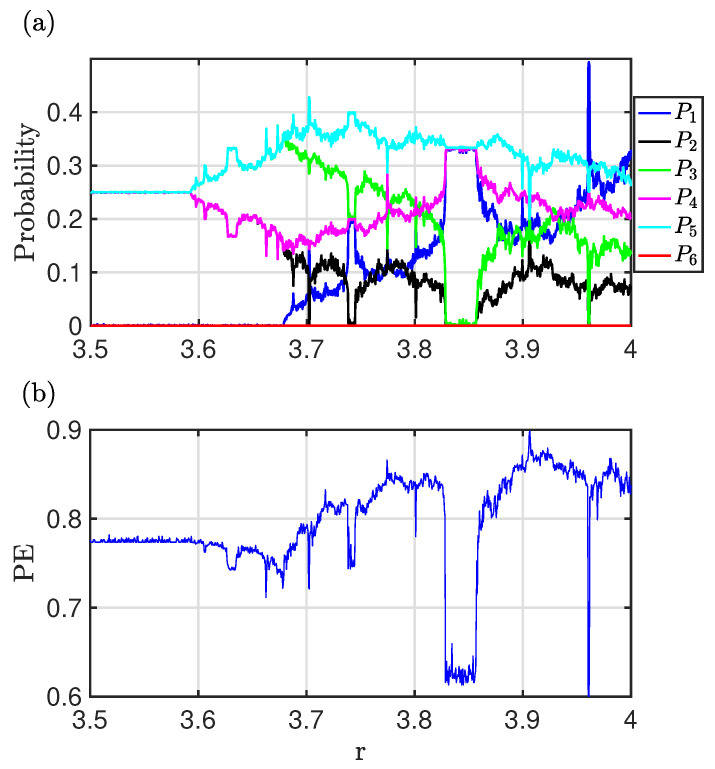
(**a**) Word populations as a function of *r* for the logistic map. (**b**) PE as a function of *r* for the logistic map. Both figures identify the windows of periodicity in this chaotic map. The word probabilities and their constraints also identify different families of chaos through their distribution and hierarchies.

Figure 1a shows the populations $P_{i}$ of the words of dimension d=3 as a function of the control parameter *r* for the logistic map ($x_{n+1}=rx_{n}\left(1-x_{n}\right),\;3.5\leq r\leq 4.0$). As *r* is varied, the system explores different chaotic regions as well as windows of periodicity. This is captured by the word populations, which identify regions with different approximate symmetries [14], characterized by different combinations of the populations $P_{i}$. These symmetries are constraints in the dynamics. The more obvious one for the logistic map is the fact that word-6 (210) is a forbidden word ($P_{6}=0$), which imposes a restriction on the populations of the other words. Another constraint in the chaotic regime 3.592<r<3.679 is that $P_{2}=P_{4}\neq P_{3}=P_{5}$ and $P_{1}=P_{6}=0$. For the region 3.679<r<3.891, the populations satisfy $P_{2}=\frac{1-3P_{1}}{2}-P_{3}$, $P_{4}=P_{1}+P_{2}$ and $P_{5}=P_{1}+P_{3}$.

### 2.2. Permutation Entropy

With the populations $P_{i}$ of the words of dimension *D* ($P_{\left[012\right]}\to P_{1}$, $P_{\left[021\right]}\to P_{2}$, …, $P_{\left[210\right]}\to P_{6}$), we compute the permutation entropy (PE) as [5]:

(1) $$PE=-\frac{\sum_{i=1}^{N}P_{i}log\left(P_{i}\right)}{log(N)}$$

Here, *N* is the number of words. This is N=D! if there are no consecutive equal values (x(i)≠x(i+1), …). In order to account for the possibility of x(i)=x(i+1), we include a seventh word for D=3, as $P_{111}$. PE is normalized so that 0≤PE≤1. For a purely random process, $P_{i}=\frac{1}{N}\;\forall i\;\Rightarrow \;PE=1$, whereas if there is only one word in the dynamics, then $P_{i^{*}}=1,\;and\;P_{i}=0\;\forall i\neq i^{*}\;\Rightarrow \;PE=0$. Figure 1b shows the permutation entropy for the logistic map as a function of the control parameter. There are sudden drops in PE when the dynamics enters in a window of periodicity, associated with sharp changes in the temporal symmetries.

### 2.3. Combined Complexity Measures

**Figure 2:**
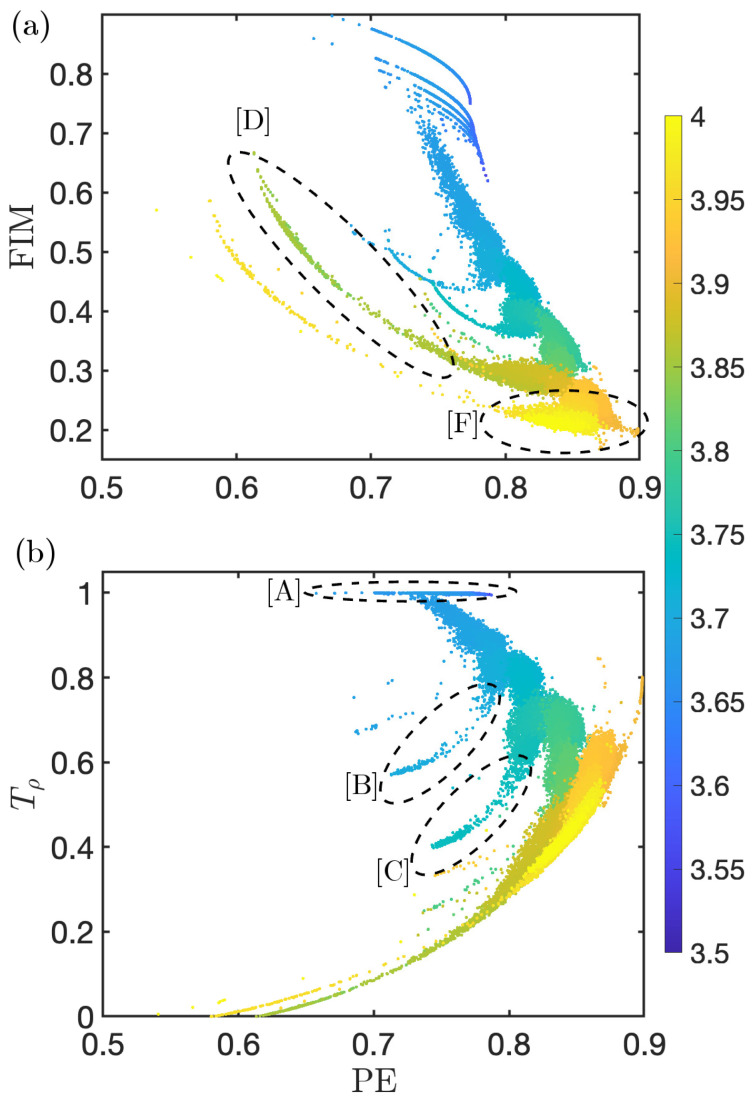
(**a**) Fisher Information Measure versus PE. (**b**) Reversibility $T_{\rho }$ versus PE. Both combinations show a clear signature that tracks the different dynamics performed by the map as the control parameter is varied. FIM computed using $SA^{*}$ sorting array: $[P_{2}$-$P_{4}$-$P_{1}$-$P_{5}$-$P_{3}$-$P_{6}]$ (see Ref. [14]). Dashed ellipses point to some families of chaos. The different circled regions indicate different families of chaos, from **[A]** laminar with more dynamical constraints to **[F]** more chaotic (details in main text).

Combining separate complexity quantifiers has been shown to help highlight differences and identify similarities shared by different dynamics and are thus excellent tools to identify and classify families of chaos. Figure 2 shows two of these combinations, Fisher Information Measure, FIM (see Appendix A for details), versus PE, and reversibility $T_{\rho }$ versus PE for the logistic map (see Refs. [13,14] for details of these quantifiers). These figures portray a 2D signature that tracks the evolution of the complexity of the dynamics as the system evolves in parameter space. The color code indicates the control parameter 3.5≤r≤4.0.

This geometric representation of the chaotic map allows us to differentiate dynamical regions. While PE quantifies the structure of the dynamics, the system can present the same PE with different FIM or $T_{\rho }$ values. Additionally, in these 2D spaces, the dynamical states cluster together and create distinct visual patterns, making it possible to identify different families of chaos (some indicated with dashed ellipses in Figure 2). It can be appreciated that for low values 3.5≤r≤3.6, the system sits in the laminar region of these 2D fingerprints, shown by the top blue lines ([A]). The system then evolves to gradually increase PE while it decreases FIM and $T_{\rho }$ (moves generally down to the right). This prevailing tendency is not uniform; it shows structure and presents escapes from it ([B], [C]). These correspond to the windows of periodicity and the period-doubling route to chaos associated to them, and can be tracked in these representations. Cluster [D] indicates the route to chaos at r≈3.83, while [F] corresponds to the more chaotic behavior close to r≲4, where the dynamics has lost most of the previous constraints, although $P_{6}=0$ still holds.

### 2.4. Universality

The 2D fingerprints observed when combining complexity quantifiers for the logistic map are not unique to this chaotic system but are universal across many chaotic iterative maps. This widespread behavior was demonstrated in Ref. [14] for FIM versus PE. Since these quantifiers are based on temporal correlations and approximate dynamical symmetries, this universality is a reflection of underlying universal dynamical symmetries. These universal features can be revealed using various complexity quantifiers that are designed to capture temporal correlations in the dynamics [5,11,12,13,16]. Each quantifier, or their combinations, extracts a specific characteristic of the dynamics, and the most suitable approach depends on the particular dynamical system.

**Figure 3:**
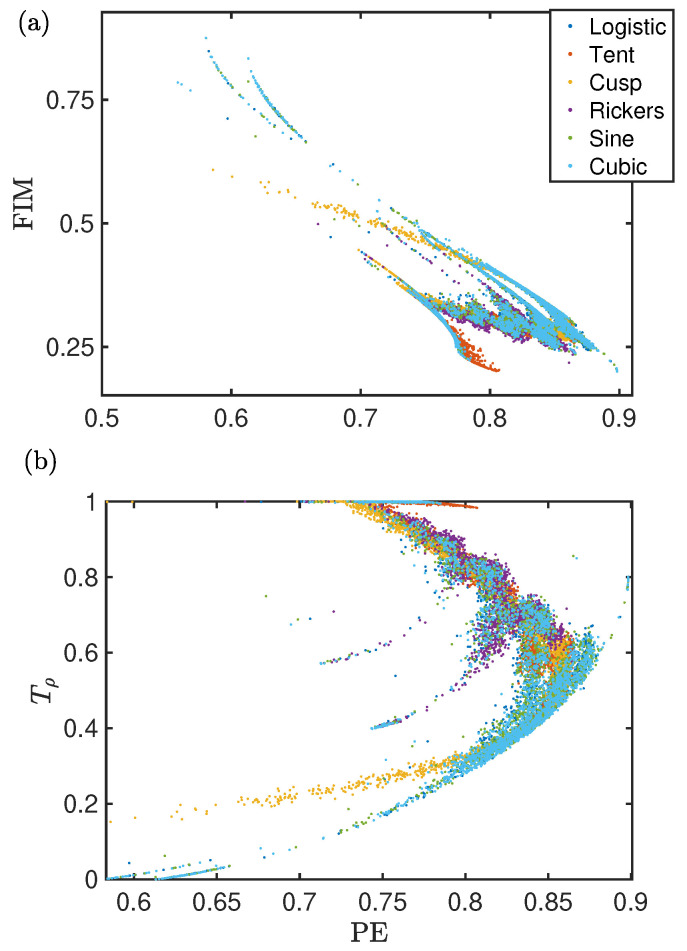
For the logistic (3.5≤r≤4.0), tent (1.1≤r≤2), cusp (1≤r≤2), Ricker’s (15≤r≤20), sine (0.85≤r≤1.00), and cubic (2.3≤r≤2.6) maps: (**a**) FIM versus PE. Sorting array for FIM: $SA^{†}$=$[P_{4}$-$P_{3}$-$P_{1}$-$P_{6}$-$P_{5}$-$P_{2}]$. (**b**) Reversibility $T_{\rho }$ versus PE.

Figure 3 shows the universality features for different chaotic maps (see Appendix A and caption for details) using the same two combinations of quantifiers as in Figure 2. Figure 3a plots FIM versus PE. FIM is a local measure, in this case computed with the populations of the words and using the sorting array $SA^{†}$ from Ref. [14]. PE is a global measure, so this Fisher–Shannon plane (see Figure 3a) combines local and global dynamics. All the maps overlap, following the same 2D pattern. The trajectory of each map’s state in this space reflects the evolution of symmetries with respect to control parameters, showing how these progressions are shared across all maps.

There are subtle differences that allow for differentiation between the maps. For example, the cusp and tent maps exhibit the least structure, as if they outline the skeleton around which other maps display their more complex features. This indicates that, while these two maps share the same dynamical features and constraints as the others because they overlap on the plane, they do not show deviations from the main path. This indicates that the sharper shape of these two maps correlates with the lack of transitions of the dynamics to regions with more constraints, which is itself related to windows of periodicity or period-doubling routes to chaos.

Figure 3b shows the TARDYS reversibility quantifier, $T_{\rho }=1-\left|P_{1}-P_{6}\right|-\left|P_{2}-P_{4}\right|-\left|P_{3}-P_{5}\right|$, plotted against PE. $T_{\rho }$ captures the reversibility of the dynamics as it weights the probabilities of self-reversible words, i.e., word 1 (012) is the reversible of word 6 (210), word 2 (021) is the reversible of word 4 (120), and word 3 (102) is the reversible of word 5 (201). Similar to Figure 3a, all the maps display the same fingerprint on the plane, and they all reveal the same families of chaos.

The quantifiers presented in Figure 3 (FIM, PE, and $T_{\rho }$) are of general relevance to any dynamical system. Other quantifiers, such as $T_{\alpha }$ or $T_{\beta }$, were defined based on the dynamical symmetries of a specific physical system, a photonic neuron [13]. It is a very specific symmetry that might be relevant to a subset of dynamical systems, although it has been found in various systems.

**Figure 4:**
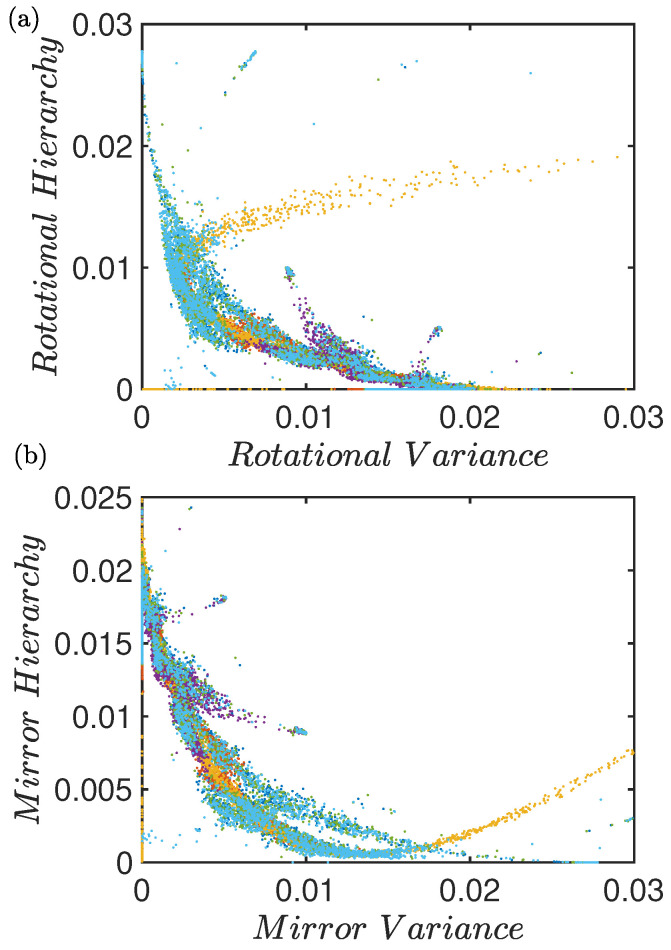
For the logistic, tent, cusp, Ricker’s, sine, and cubic maps: (**a**) Rotational Hierarchy versus Rotational Variance [12]. (**b**) Mirror Hierarchy versus Mirror Variance. Legend as in Figure 3.

Because these inherent features originate in internal temporal symmetries, other combinations of quantifiers can express more meaningful and useful representations. Portraying other quantifiers that highlight generic dynamical features such as Rotational and Mirror symmetries can be more significant. In Figure 4, we combine these symmetry quantifiers. As expected, they display the universality present in these chaotic systems. Tracking their position in this 2D space can tell us how each of these two specific symmetries evolve with the control parameter, but also how they contribute to the overall change in dynamical symmetry.

Figure 4a shows Rotational Hierarchy versus Rotational Variance (quantifiers defined in Appendix [12]). They measure the rotational symmetry present in the dynamics through the sets of words (012, 120, 201) and (021, 102, 210). The fingerprint of all the maps lie on top of each other, indicating the universality of the temporal dynamics. As with previous combinations of quantifiers (Figure 3), some maps show a more detailed structure, making this a differentiating trait. Again, the cusp and the tent maps show less detail in this fingerprint and they define the main skeleton area that all the other maps fall into.

Figure 4b shows Mirror Hierarchy versus Mirror Variance for the set of chaotic maps. Similar observations as Figure 4a can be made for this other dynamical symmetry. Each combination of quantifiers draws a different characteristic fingerprint as each one depicts a particular aspect of the global symmetry of the dynamical system, but all show the universality pattern and capture transitions in dynamics that allow us to classify families of chaos.

All of these results suggest that this universal behavior can be emphasized, better represented, and interpreted if we define a suitable symmetry space that accentuates the dynamical symmetries embedded in the temporal correlations of words of dimension D=3.

## 3. Symmetry Space and Universality

To capture the essence of the temporal approximate symmetries in a dynamical system—and, as we see below, to gain an extra dimension in representation given the relationship of the length of the vector to the PE—we use a new entropy sub-channel space where the dynamics is represented by a vector $\vec{\Phi }$ whose length corresponds to PE, and its components $\left({\vec{\phi }}_{1},{\vec{\phi }}_{2},{\vec{\phi }}_{3}\right)$ relate to approximate reversible symmetries in the dynamics. This technique is introduced in Ref. [15], where it is applied to the Duffing oscillator as a paradigmatic flow displaying chaos where the value of being able to see multiple regimes of dynamical behavior in this compact manner is discussed in detail. The method pairs the populations of the words that are time-reversible with each other, such as [012,210], [021,120], and [102,201], into vectors as follows:

(2) $${\vec{\phi }}_{1}=\left(\begin{array}{c} \pi_{1}\\ \pi_{6}\\ \frac{S_{111}}{\sqrt{3}} \end{array}\right);\;{\vec{\phi }}_{2}=\left(\begin{array}{c} \pi_{2}\\ \pi_{4}\\ \frac{S_{111}}{\sqrt{3}} \end{array}\right);\;{\vec{\phi }}_{3}=\left(\begin{array}{c} \pi_{3}\\ \pi_{5}\\ \frac{S_{111}}{\sqrt{3}} \end{array}\right)$$

where

(3) $$\pi_{i}=\sqrt{-\frac{P_{i}\;log\left(P_{i}\right)}{log(N)}}$$

and

(4) $$\pi_{111}=\sqrt{-\frac{P_{111}\;log\left(P_{111}\right)}{log(N)}},$$

i.e., $P_{111}$ quantifies the population of words that contain equal values, i.e., x(i)=x(i+1), or x(i+1)=x(i+2), or x(i)=x(i+2), or x(i)=x(i+1)=x(i+2).

These three vectors ${\vec{\phi }}_{i}$ define a coordinate system where each axis identifies the relevance of a particular reversibility constraint. We then create a symmetry vector as follows:

(5) $$\vec{\Phi }=\left(\begin{array}{c} |{\vec{\phi }}_{1}|\\ |{\vec{\phi }}_{2}|\\ |{\vec{\phi }}_{3}| \end{array}\right)$$

where by construction this vector captures several aspects of the dynamics of the system: its magnitude, $\left|\vec{\Phi }\right|$, quantifies the total amount of temporal symmetries present while its components indicate the significance of each symmetry within the dynamical system.

**Figure 5:**
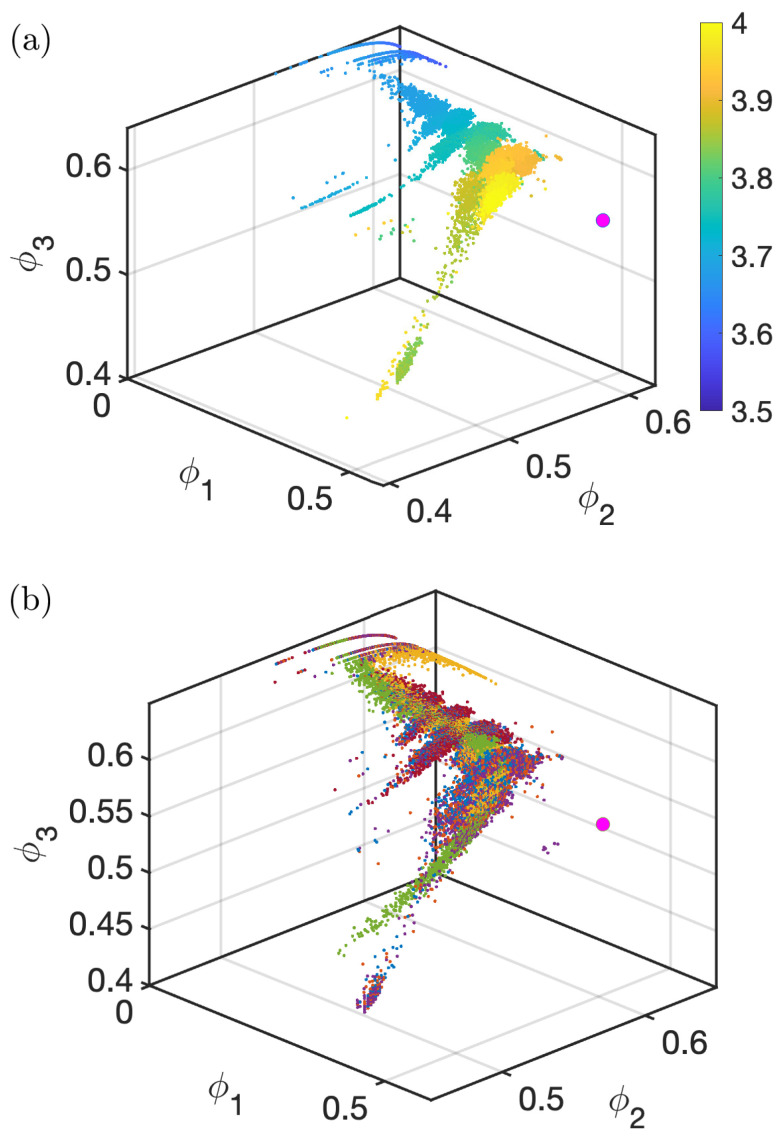
(**a**) Symmetry vector plotted in symmetry space for the logistic map. Color code indicates the control parameter 3.5≤r≤4.0. The pink dot refers to a random dynamics. (**b**) Symmetry vector for several chaotic iterative maps. Colors as in Figure 1.

Figure 5a plots the symmetry vector in symmetry space for the logistic map, where the color indicates the control parameter *r*. The projection of this vector tracks the evolution of the chaotic map as the control parameter runs in the 3.5≤r≤4.0 range. The fingerprint on this space shows how the symmetries of the dynamics evolve.

The system starts without the presence of the symmetry $\phi_{1}$. It evolves in a pseudo-laminar regime where $\phi_{2}$ and $\phi_{3}$ progress on a curve while $\phi_{1}$ remains unchanged. Then, there is a transition where $\phi_{1}$ starts to gain relevance while the other two symmetries stay mostly unchanged except for some drastic escapes to lower values. These departures of $\phi_{2}$ and $\phi_{3}$ in this range are related to the period-doubling routes to chaos and the windows of periodicity. When the system approaches the most chaotic regime, $\phi_{2}$ and $\phi_{3}$ decrease while $\phi_{1}$ stays almost constant again.

The magenta dot in the figure indicates a state for which the dynamics is random. Because all the words are equally probable at this point, the ordinal dynamics here is maximally reversible ($P_{1}=P_{2}=\ldots =P_{6}=\frac{1}{6}$). The logistic map approaches that randomness dot as it gets more chaotic, but the remaining constraints (such as $P_{6}=0$) prevent it from reaching this point.

Figure 5b shows the universal character of the symmetry vector among the different chaotic iterative maps. $\vec{\Phi }$ also captures how all maps lie on top of the same fingerprint in symmetry space. This highlights the fact that this universality feature, as well as those previously found elsewhere, is a symmetry universality, embedded in the temporal dynamics of these systems.

Not only does this combination of constraints expressed through the word populations quantify the presence of reversibility in the dynamics and the contribution of each temporal symmetry, but, defined this way (Equations (3) and (5)), the magnitude of this vector is equivalent to the permutation entropy of the time series of the dynamics. $\pi_{i}$ can be thought of as a sub-entropy, or the *i*-component of an entropy vector such that ${\left|\vec{\Phi }\right|}^{2}=PE$.

It is worth noting here the relevance of this symmetry vector as a three-component object, whose components contain specific information about different but complementary dynamical symmetries. Despite the fact that $\sum_{i}^{N}P_{i}=1$, the length of this vector changes with the control parameter, as it depends on the presence of temporal symmetries. For some systems, this vector will move in a subspace of this symmetry space, restricting its values to a plane, indicating that the symmetry perpendicular to this plane remains unaltered, even though the other symmetries change in non-trivial manners (see Ref. [15]).

## 4. Universality Using Four-Dimensional Words

**Figure 6:**
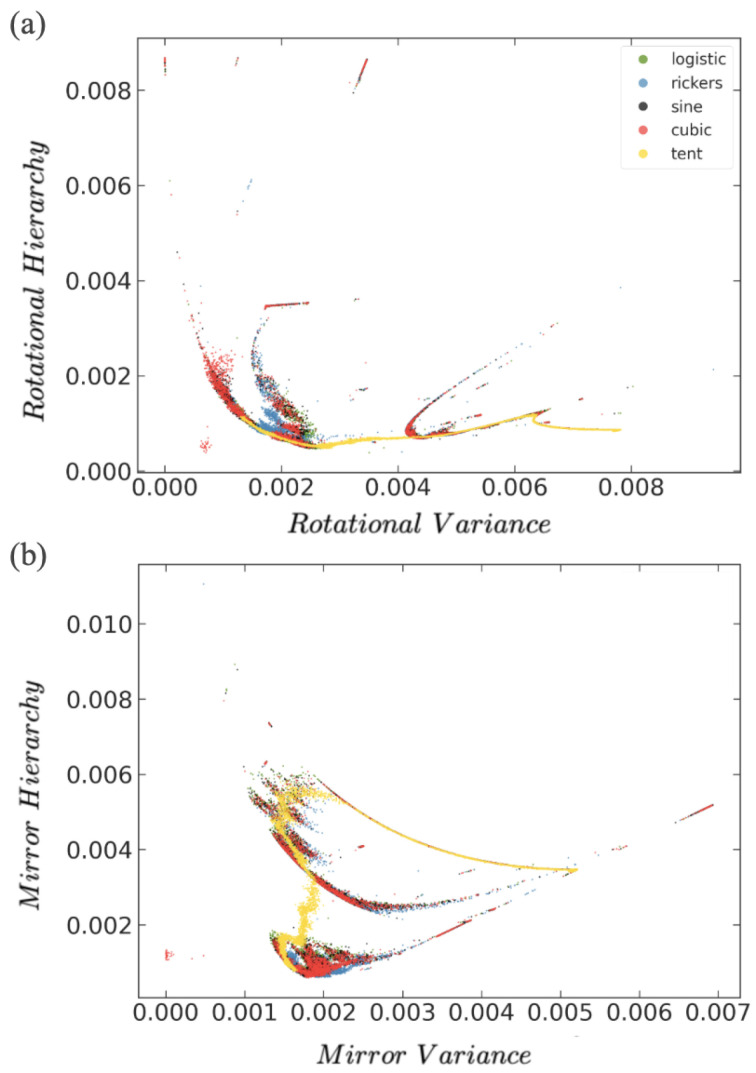
(**a**) Rotational Hierarchy versus Rotational Variance for several 1D iterative maps for words of length 4. (**b**) Mirror Hierarchy versus Mirror Variance for words of length 4.

At this point, one could argue that if exploring words of higher length, *D* would be convenient as the possibilities of patterns increase considerably and we could study more complex symmetries. Also, computing higher-length words would involve longer temporal correlations and, therefore, they explore the longer memory of the system. To investigate this, we compute the symmetry quantifiers for words of length D=4 (Figure 6). They show interesting fingerprints when pairing different quantifiers, and we also see the universality patterns at this temporal scales. Figure 6a plots a generalization of Rotational Hierarchy versus Rotational Variance for D=4 for the logistic map. For D=3, we have two rotation groups: (012,120,201) and (021,210,102). For D=4, we have six rotational groups: (0123,3012,2301,3012), (0132,1320,2013,3201), etc. As we see, Figure 6a clearly distinguishes families of chaos in the well-defined and differentiated trajectories that the dynamics follows.

Figure 6b plots a generalization of Mirror Hierarchy versus Mirror Variance for words of D=4 for different chaotic iterative maps. This combination shows a different 2D fingerprint but still identifies the different dynamical regions. As found previously, all maps share the same 2D fingerprint, revealing that universality is also present at this level of depth in words length. As shown in Figure 3, Figure 4 and Figure 5, for words of D=3, the tent and cusp maps have the least structure and define the skeleton around which the other maps are deployed, demonstrating it as good tool to identify not just the commonalities among maps but also their differences.

Despite the richness of possibilities for longer-length words, understanding the meaning of the symmetries is less intuitive and not always possible to generalize. Also, because of the high number of possible words (24 for D=4), it is not trivial to find the suitable combinations equivalent to the D=3 symmetries, or to define a three-dimensional symmetry vector space. Creating a Φ-space for D=4 would result in a 12-dimensional space (12 pairs of reversible words), unless alternative multi-word combinations are employed, which are not immediately straightforward or intuitive. Furthermore, analysis of higher-length words imposes a statistical challenge as much longer time series are required, which is not always feasible from an experimental point of view.

## 5. Conclusions

We have investigated the temporal correlations within the nonlinear dynamics of iterative chaotic maps, focusing in particular on the universality of temporal symmetries in these systems. To achieve this, we have employed established combinations of temporal dynamical quantifiers, such as permutation entropy, Fisher Information Measure, and TARDYS quantifiers, along with new combinations that incorporate Mirror and Rotational symmetries.

Additionally, we have used a novel technique that projects the system’s dynamics into a symmetry vector space. This symmetry space is constructed using entropy sub-channels, which are combined into three axes that quantify the reversibility of the dynamics. The position of a dynamical system in this symmetry vector space provides insights into the system’s overall temporal symmetry, while its evolution with the control parameter reveals how these symmetries vary in chaotic regimes. The magnitude of this vector, $\left|\vec{\Phi }\right|$, corresponds to the system’s permutation entropy.

Our results reveal a universal pattern in the temporal correlations across all the chaotic systems studied. This pattern emerges regardless of the specific combination of complexity quantifiers used. While each combination retains its own distinct signature, the chaotic maps share a consistent “fingerprint” in every representation space.

More significantly, we linked these universal features to specific temporal and reversible symmetries, which are captured within the permutation entropy. The construction of $\vec{\Phi }$ as a merger of sub-entropies combines the power of entropy as a complexity quantifier with the temporal symmetries of the dynamics, in particular with the reversibility symmetry of the system.

Not only do these techniques allow us to identify similarities and differences across chaotic dynamics, classify families of chaotic behavior, and uncover universal patterns, but they also connect these patterns to the system’s intrinsic dynamical symmetries. Moreover, for chaotic systems exhibiting this universality, their evolution in symmetry space enables us to track where the system originates and where it is heading in the parameter space, offering potential for forecasting dynamic transitions.

The concept of universality in chaotic maps is not new, with roots tracing back to Feigenbaum’s 1976 discovery of universality constants in the bifurcation diagram of the logistic map. However, the universal signature we present here pertains to the symmetries in the temporal correlations of chaotic map time series, marking a novel perspective on the universality of chaotic systems.

We have used a diverse set of complexity measures to identify the shared underlying traits in the chaotic maps studied, and to uncover their relation to dynamical symmetries. The techniques presented in this paper provide complementary information and strong evidence of the universality features that all these maps share, while they are not computationally demanding. Future research could focus on finding a deeper, potentially analytical demonstration of these generalized patterns of universality.

## Data Availability

The data that support the findings of this study are available from any of the authors upon reasonable request.

## Appendix A

### Appendix A.1. Rotational and Mirror Quantifiers

The Rotational and Mirror quantifiers used in Figure 4 and Figure 6 measure the symmetries of words that are rotations of each other or mirror images of each other [12]. These are contained in subgroups of words. Those words that are obtained from another word through a rotation can be grouped as R1: 012-120-201 and R2: 021-210-102. Pairs of words that are mirror images can be groups as M1: 012-210, M2: 021-120, and M3: 102-201. We define the quantifiers as follows:

- Rotational Variance:
  (A1) $$\psi =\frac{{\left(P_{1}-P_{R1}\right)}^{2}+{\left(P_{4}-P_{R1}\right)}^{2}+{\left(P_{5}-P_{R1}\right)}^{2}+{\left(P_{6}-P_{R2}\right)}^{2}+{\left(P_{2}-P_{R2}\right)}^{2}+{\left(P_{3}-P_{R2}\right)}^{2}}{6}$$
- Rotational Hierarchy:
  (A2) $$\Psi =\frac{{\left(P_{R1}-P_{all}\right)}^{2}+{\left(P_{R2}-P_{all}\right)}^{2}}{2}$$
- Rotational Variance:
  (A3) $$\xi =\frac{{\left(P_{1}-P_{M1}\right)}^{2}+{\left(P_{6}-P_{M1}\right)}^{2}+{\left(P_{2}-P_{M2}\right)}^{2}+{\left(P_{4}-P_{M3}\right)}^{2}+{\left(P_{3}-P_{M3}\right)}^{2}+{\left(P_{5}-P_{M3}\right)}^{2}}{6}$$
- Rotational Hierarchy:
  (A4) $$\Xi =\frac{{\left(P_{M1}-P_{all}\right)}^{2}+{\left(P_{M2}-P_{all}\right)}^{2}+{\left(P_{M3}-P_{all}\right)}^{2}}{3}$$

where

(A5) $$P_{R1}=\frac{P_{1}+P_{4}+P_{5}}{3};\;P_{R2}=\frac{P_{6}+P_{2}+P_{3}}{3};$$

(A6) $$P_{M1}=\frac{P_{1}+P_{6}}{2};\;P_{M2}=\frac{P_{2}+P_{4}}{2};\;P_{M3}=\frac{P_{3}+P_{5}}{2}$$

and

(A7) $$P_{all}=\frac{P_{1}+P_{2}+P_{3}+P_{4}+P_{5}+P_{6}}{6}$$

### Appendix A.2. Fisher Information Measure

Fisher Information Measure (FIM) [17,18] is a complexity measure of the gradient content of a distribution. It can describe the evolution of physical systems and characterize complex signals, either as an alternative to Shannon entropy or as a complementary tool. FIM quantifies differences between neighboring values in a time series, effectively acting as a measure of the local derivative of the series. For a distribution of probabilities $p_{i}$, it can be defined as follows:

(A8) $$FIM=F_{0}\sum_{i=1}^{N-1}{\left({\left(p_{i+1}\right)}^{1/2}-{\left(p_{i}\right)}^{1/2}\right)}^{2}\;,$$

where $F_{0}=1$ if $p_{i^{*}}=1$ for $i^{*}=1$ or $i^{*}=N$, and $p_{i}^{*}=0\;\forall i\neq i^{*}$; otherwise, $F_{0}=\frac{1}{2}$. This measure depends on the ordering of the probabilities $p_{i}$, as was discussed in Ref. [14], but the overall behavior is captured by all sorting arrays.

Vignat and Bercher [16] introduced the Fisher–Shannon information plane to use the complementarity aspects of both measures as an effective method to characterize the non-stationary behavior of a complex signal.

### Appendix A.3. Chaotic Maps

The 1D chaotic iterative maps studied in this paper are as follows:

- Logistic map: $x_{i+1}=rx_{i}\left(1-x_{i}\right)$, for 3.5≤r≤4.
- Tent map: $x_{i+1}=r\;min\left\{x_{i},1-x_{i}\right\}$, for 0≤r≤1.
- Cusp map: $x_{i+1}=1-r\sqrt{|x_{i}|}$, for 0≤r≤1.
- Ricker’s population model: $x_{i+1}=rxe^{-x_{i}}$, for 15≤r≤20.
- Sine map: $x_{i+1}=r\;sin\left(\pi x_{i}\right)$, for 0.85≤r≤1.
- Cubic map: $x_{i+1}=rx_{i}{\left(1-x_{i}\right)}^{2}$, for 2.3≤r≤2.6.
